# Paraneoplastic Opsoclonus-Myoclonus Syndrome as a Rare Presentation of Small-Cell Lung Cancer

**DOI:** 10.7759/cureus.32066

**Published:** 2022-11-30

**Authors:** Inês Moreira, Isabel Vilas-Boas, Maria Cassiano Neves

**Affiliations:** 1 Medical Oncology, Portuguese Institute of Oncology Francisco Gentil, Porto, PRT

**Keywords:** small-cell lung cancer, immune-mediated, paraneoplastic syndromes, kinsbourne syndrome, opsoclonus-myoclonus syndrome

## Abstract

Opsoclonus-myoclonus syndrome (OMS), also known as Kinsbourne syndrome or dancing eyes syndrome, is an extremely rare neurological condition that comprises a heterogenous constellation of symptoms including opsoclonus along with diffuse or focal body myoclonus. It is usually referred to as a paraneoplastic entity, but it may also be associated to an infectious, metabolic, or idiopathic cause. Small-cell carcinoma of the lung is the most commonly reported malignancy associated with OMS. The authors describe a case of a 69-year-old male that presented with ataxic gait, phono- and photophobia, vertigo, dizziness, lethargy, nausea, and vomiting. During examination, rapid, multidirectional eye movements; slight dysarthria; and facial myoclonus were noted. He was admitted to the hospital, and after a thorough study, a diagnosis of OMS was established. Intravenous corticosteroids were started, alongside physiotherapy, and a slight improvement of his symptoms was noted. Imaging revealed a suspicious lesion in the left lung, along with lymphadenopathies and bone metastases. Histology confirmed the diagnosis of stage IV small-cell lung cancer (SCLC). Chemotherapy (ChT) with carboplatin and etoposide was started, and a gradual improvement of his neurological complaints was noted. After six cycles, the disease progressed, and second-line ChT with topotecan was started. After two cycles, the patient experienced significant clinical deterioration and eventually died. In conclusion, OMS is a poorly understood condition with uncertain neurological prognosis. The treatment of the primary neoplasm may improve neurological symptoms. The recognition of paraneoplastic syndromes is of utmost importance since early diagnosis of a malignancy relates to better outcomes.

## Introduction

Paraneoplastic neurological syndromes can affect any part of the nervous system and are a rare complication of malignancy, occurring in less than 1% of cancer patients [1]. Opsoclonus-myoclonus syndrome (OMS), also known as Kinsbourne syndrome or dancing eyes syndrome, is an extremely rare neurological condition that is still poorly understood. It comprises a heterogenous constellation of symptoms that include opsoclonus along with diffuse or focal body myoclonus (nonepileptic involuntary muscular jerks). Opsoclonus consists of involuntary, irregular, high-frequency oscillations of the eyes with multidirectional saccades [2]. Additional clinical features such as ataxia, tremors, vertigo, dysarthria, sleep disturbances, encephalopathy, and cognitive and psychiatric symptoms are usually observed [2,3].

OMS is better described in children and generally associated with neuroblastoma [2,4], but most of the literature regarding this condition in adults is largely confined to case reports and small case series. As for its etiology in adults, it is usually referred to as a paraneoplastic entity, but it may also be associated to an infectious, metabolic, or idiopathic cause [2]. Similar to other paraneoplastic syndromes, an immune-mediated mechanism triggered by the tumor microenvironment is believed to play a role in its genesis, and the presence of anti-Ri, anti-Yo, and anti-Hu antibodies in the serum or cerebrospinal fluid has been described [2,5]. This immune response is likely initiated against the neuronal antigens expressed by the tumor. Nonetheless, the majority of patients with OMS are seronegative for antineuronal antibodies, despite methodological limitations being a possible contributor [2].

Small-cell carcinoma of the lung is the most commonly reported malignancy associated with OMS in adults, but cases related to breast cancer, melanoma, and gynecologic and urogenital cancers have been reported [3,5-11]. Importantly, the onset of OMS generally precedes other clinical manifestations related to cancer, offering the opportunity for an early diagnosis [8]. Hence, patients presenting with this cluster of symptoms should be evaluated for an underlying malignancy. The authors hereby present a case of a small-cell lung cancer (SCLC) patient that presented with OMS.

## Case presentation

A 69-year-old male, heavy smoker (100 pack-years), that used to work in a paint factory, with previous medical history of hypertension and ischemic stroke without sequelae, presented to the emergency room with ataxic gait, phono- and photophobia, vertigo, dizziness, lethargy, nausea, and vomiting that began a few years earlier and progressively worsened. On admission, rapid, multidirectional eye movements; slight dysarthria; and facial myoclonus were noted. Laboratory workup for complete blood count, electrolytes, and renal and hepatic function was normal. Cerebral enhanced computed tomography (CT) scan at admission did not show any relevant acute changes.

He was admitted to the hospital with suspicion of an ischemic stroke of the cerebellum. Cerebral enhanced magnetic resonance imaging (MRI) was performed that showed no acute alterations and no evidence of cerebral or cerebellar stroke or hemorrhage. An echocardiogram did not find any changes, and a Doppler ultrasound of the carotid and vertebral arteries revealed calcifications at the bifurcation of the common carotid artery bilaterally, the largest measuring 11 mm and located at the emergence of the right internal carotid artery, but no evident stenosis was found. Cerebrospinal fluid analysis was unremarkable, with microbiology and cytology being negative. Serum viral serology and antineuronal antibodies were also negative.

**Figure 1:**
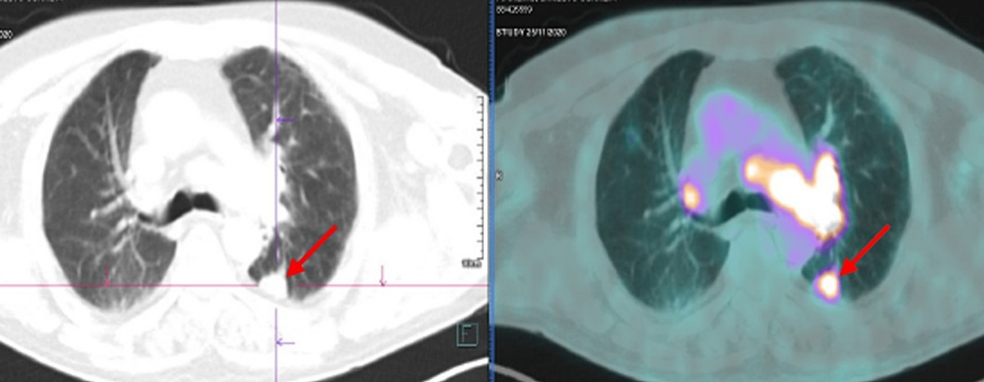
PET-CT scan showing a mass in the superior segment of the inferior lobe of the left lung (arrows) and left hilar lymphadenopathies/conglomerates. PET: positron emission tomography; CT: computed tomography

After 10 days in the hospital, the patient did not show any improvement, and a diagnosis of OMS was suspected. He began treatment with intravenous corticosteroids alongside physiotherapy, and a slight improvement of his symptoms was noted, but he still only could walk small distances and with the help of others, maintaining an ataxic gait and dizziness. To search for a paraneoplastic etiology, a positron emission tomography (PET)-CT scan of the chest, abdomen, and pelvis was ordered, which showed a 22 mm mass in the superior segment of the inferior lobe of the left lung and a left hilar conglomerate (Figure 1).

**Figure 2:**
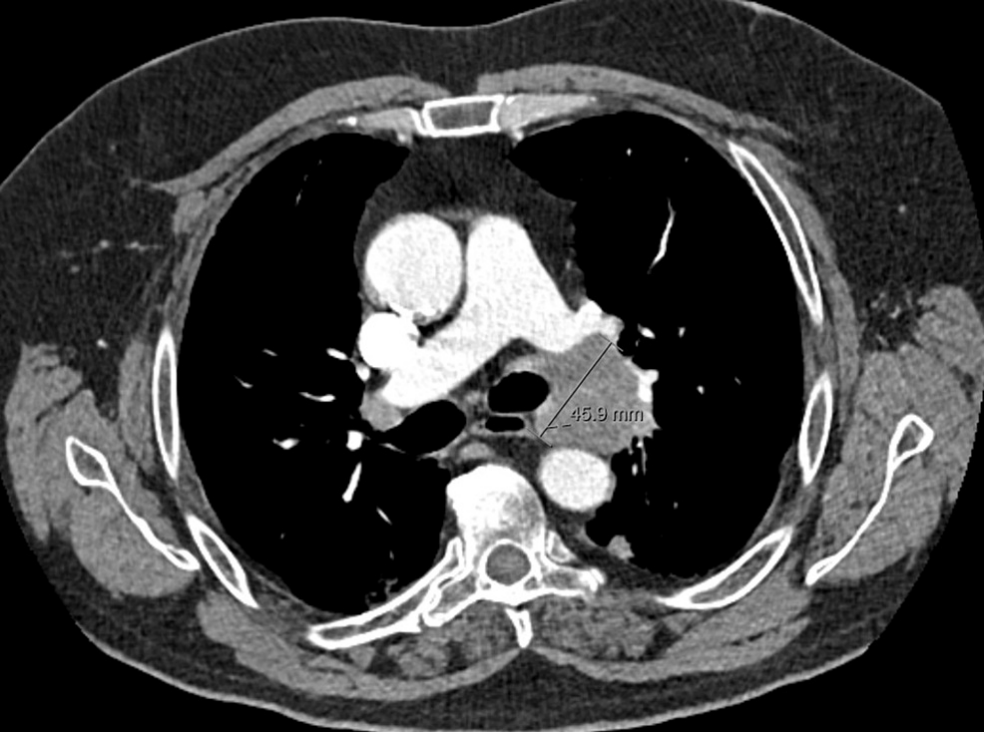
CT scan of the chest after first-line ChT, showing disease progression at the level of the left hilar lymph node conglomerate. CT: computed tomography; ChT: chemotherapy

A biopsy was performed, and histologic analysis of the lesion confirmed the diagnosis of small-cell lung cancer (SCLC). The PET-CT scan also showed a bone metastasis in the third cervical vertebrae in addition to the lung neoplasm and lymphadenopathies. The disease was staged as cT1N2M1. It was decided to treat the patient with chemotherapy (ChT) with carboplatin and etoposide. He experienced a gradual improvement of his neurological complaints and, after two cycles, was able to walk small distances without help and was capable of performing his own hygiene, allowing for steroid weaning, although still maintaining slight horizontal eye movements and vertigo. A thoracic CT scan after four cycles showed stable disease. The patient completed six cycles of ChT and remained under close observation afterward. A CT scan performed one month after the sixth cycle revealed disease progression, with an increase in size of the hilar conglomerate (Figure 2).

At this point, the patient was experiencing de novo headaches and important fatigue and being more dependent for daily activities. A cerebral enhanced MRI was ordered, and a single small left cortical parietal lesion was described, with no cerebral edema or mass effect. He started second-line ChT with topotecan. The case was discussed in the multidisciplinary tumor board, and due to the patient’s frailty, it was decided to repeat the MRI after three cycles and not to start cerebral radiotherapy at the moment.

After two cycles, the patient experienced significant clinical deterioration. His dyspnea also aggravated, and he was then dependent of continuous oxygen therapy. ChT was interrupted and best supportive care proposed. Two months after the last cycle, he further deteriorated, was admitted to the hospital for symptom control, and died shortly after. This patient was diagnosed and treated before the approval of immunotherapy by the Portuguese regulatory agency in this setting, and that is why it was not prescribed.

## Discussion

Lung cancer remains the most common malignancy after non-melanocytic skin cancer and is the leading cause of cancer mortality worldwide. Primary lung cancer is usually divided into two groups: non-small-cell lung cancer, accounting for 80%-90% of lung cancer cases, and SCLC, accounting for the remaining 10%-20% [12]. SCLC is strongly associated to smoking and is described as the most aggressive form of lung cancer, characterized by a rapid growth and also by a rapid response to ChT and sensitivity to radiotherapy, with advanced disease being common at diagnosis [13]. However, due to early treatment resistance, a high risk of recurrence and poor response to second-line therapies, five-year overall survival (OS), is less than 10%; nonetheless, recently approved immunotherapy drugs, such as atezolizumab or durvalumab, in combination with ChT and then used as maintenance treatment allowed for an increase in OS in the metastatic setting [13].

SCLC is one of the most frequent malignancies associated with paraneoplastic syndromes. These are usually related to abnormal hormone production by neoplastic cells, such as ectopic Cushing’s syndrome or hyponatremia of malignancy, or to immune-mediated neurological syndromes. The neurological paraneoplastic syndrome most frequently associated with SCLC is Lambert-Eaton myasthenic syndrome, present in 1%-3% of patients, with OMS occurring in less than 1% [14].

The best treatment approach for OMS is still debatable, and evidence-based recommendations for its management are limited. When an underlying cause is identified, treatment should be directed to that cause. If OMS emerges as a paraneoplastic syndrome, the diagnosis and treatment of the underlying malignancy remain the main step in its management [2]. Since OMS seems to result from an autoimmune process, the use of immunosuppressive agents such as corticosteroids, intravenous immunoglobulin, mycophenolate mofetil, azathioprine or rituximab, plasmapheresis, or a combination of these therapies could also be considered [2,4]. Due to the rarity of OMS, outcome-related data is scarce, but improvement and even recovery have been reported, and the early initiation of treatment appears to be related to improved neurological outcomes [4]. Idiopathic OMS tends to occur in younger patients and have a better response to immunosuppressive agents and a milder clinical evolution, whereas paraneoplastic OMS seems to be more severe, with worse response to treatment [15].

In this case, the patient experienced an improvement in his OMS-related complaints after the beginning of corticosteroids and physiotherapy but mainly after the start of ChT. This may be due not only to the antineoplastic effect of these drugs but also to their immunosuppressive effects. As previously mentioned, antineuronal antibodies are not frequently identified, and such was the case with this patient. Although SCLC usually has a good response to first-line ChT, this patient only experienced stable disease as best response. Despite the initial improvement of symptoms, when the cancer progressed, the patient clinically deteriorated and eventually died. The early recognition and diagnosis of paraneoplastic neurological syndromes are therefore of the outmost importance, not only to allow for an early detection of an underlying malignancy but also to improve the chance of better neurological outcomes by starting treatment as soon as possible.

## Conclusions

OMS is a poorly understood condition with an uncertain neurological prognosis. Due to its rarity, a high degree of suspicion is required for diagnosis, and evidence-based recommendations for its management are limited. The recognition of paraneoplastic syndromes is of utmost importance since the early diagnosis of a malignancy relates to better outcomes. The treatment of the primary neoplasm may improve neurological symptoms.
